# Successful implementation of a medical emergency team: 2-year experience in a teaching hospital

**DOI:** 10.1186/cc14490

**Published:** 2015-03-16

**Authors:** A Tridente, J Elmore, R Varia, T Mahambrey

**Affiliations:** 1 St Helens and Knowsley, Liverpool, UK

## Introduction

A medical emergency team (MET) was introduced in our institution in January 2012 to provide timely response to the needs of acutely ill inpatients and cardiac arrest calls. The MET assesses the patient and prescribes a management plan for the responsible team to follow; promptly stabilising and transferring patients to a place of safety where required. We aimed at evaluating the effects of introducing the MET on clinically relevant processes and outcomes.

## Methods

Prospective data were analysed using STATA 10.1. The primary outcome measure was immediate mortality (defined as mortality at conclusion of the MET intervention); the secondary outcome measures were admission to critical care after a MET call and cardiac arrest.

## Results

A total of 5,763 MET calls were made between 9 January 2012 and 4 March 2014, of which 5,310 (92.1%) were MET calls, 349 (6.1%) cardiac arrest calls, 36 (0.6%) false alarms and 68 (1.2%) unclassified. The number of calls increased by 32.7% from 2,255 in 2012 to 2,993 in 2013, with all month-specific comparisons showing significant increases in MET activity (ranging from 0.5% to 103.6% increases). MET activity displayed cyclical yearly changes, with the winter months and the month of August (junior doctors' changeover period) being particularly busy. Median response time (interquartile range) was 1 (1 to 2) minutes, with 99.1% calls attended to within 3 minutes. There were 210 (3.64%) immediate deaths (with no significant differences between years), 112 (1.9%) patient transfers to critical care, 233 (4%) patients were transferred to other locations (other than critical care) while 4,697 (81.5%) patients remained on the ward of origin. In 408 cases (7.1%) a do-not-resuscitate order was instituted. On multiple logistic regression analyses, when the type of call was taken into consideration, the response time had no influence on primary (mortality OR = 0.83, 95% CI = 0.63 to 1.09, *P *= 0.18) and secondary outcomes (admission to critical care OR = 0.85, 95% CI = 0.62 to 1.17, *P *= 0.33; subsequent cardiac arrest OR = 0.57, 95% CI = 0.27 to 1.2, *P *= 0.14).

## Conclusion

The MET has been successfully implemented, with demand for its services having increased by 32.7% in 1 year. The unadjusted immediate mortality rate of patients for whom a MET/cardiac arrest call is activated is 3.64%. Response time had no influence on mortality, most probably due to the rapid response time. Immediate mortality was low, probably as a result of early adequate intervention. Further evaluation of overall hospital mortality is warranted for future studies.

